# Cone penetration index for soil behaviour type prediction

**DOI:** 10.1038/s41598-022-15994-x

**Published:** 2022-07-20

**Authors:** Denilson José Ribeiro Sodré

**Affiliations:** Faculty of Civil Engineering, Federal University of Pará, UFPA, Belém, Pará, Brazil

**Keywords:** Engineering, Civil engineering

## Abstract

Several approaches have been proposed to classify soil types using both cone and piezocone penetration tests. The chart relating the normalized cone resistance (*Q*_*tn*_) and the friction ratio (*F*_*r*_) has proven to be the most reliable method. However, its practical use requires function fitting, where the chart is described by means of a soil behaviour type index. The currently available indexes for this chart, based on concentric circles and hyperbolas, lead to significant errors. Thus, to properly represent the *Q*_*tn*_*–F*_*r*_ chart, this study proposes new soil behaviour type indexes based on an analytical approach from a concentric logarithmic spiral approximation and a numerical approach from an exponential function approximation. The new indexes provide an improvement in the reproduction of the soil type zones while preserving the robustness of the original method. A discussion on the cone penetration test-based soil type classification is presented, along with a case example comparing the soil behaviour indexes in soil profiling.

## Introduction

Theoretical and experimental developments in the cone penetration test (CPT) and the piezocone penetration test (CPTu) have provided high applicability to a broad range of geotechnical properties, making them valuable tools for assessing soil type and state. As the cone responds to the in situ mechanical behaviour of the soil, the soil types are classified into groups that exhibit similar mechanical behaviour, rather than physical properties, as conventionally determined in laboratory soil testing. Thus, the term ‘soil behaviour type’ (SBT)^1^ starts to be used to describe the soil type interpretations based on CPT, emphasizing that they are not expected to be predictive of soil type identification but of the soil behaviour type.

Soil type classification methods based on CPT and CPTu are usually graphically expressed. From an extensive CPT database, Robertson^2,3^ developed a consistent chart relating the normalized cone resistance (${Q}_{tn}$) and the friction ratio (${F}_{r}$), which has become popular worldwide and has proven to be the highest quality method. As a graphical method, it cannot readily be employed in practice. It is used by means of the soil behaviour type index, ${I}_{c}$, proposed by Robertson and Wride^4^ and updated by Robertson^3^, where the chart is represented as concentric circles. However, despite being extensively used, this index diverges significantly from the chart. Schneider et al.^5^ defined a soil behaviour type index that relates ${Q}_{tn}$ and ${F}_{r}$ in terms of hyperbolic functions; however, this also significantly differs from the Robertson^2,3^ soil type zones^6^. Thus, to properly reproduce the ${Q}_{tn}$–${F}_{r}$ chart, new soil behaviour type indexes are proposed herein based on an analytical approach from a concentric logarithmic spiral approximation and a numerical approach from an exponential function approximation.

The quality of soil behaviour type prediction methods depends on the manner in which the effects of soil behaviour on cone penetrometer measurements are considered. Therefore, a thorough investigation of parameter correction and stress normalization is presented to support a discussion of cone penetrometer-based soil type classification methods. An example of a soil profile obtained from the proposed indexes and the ${I}_{c}$ index^3,4^ is provided.

## Correction of cone penetrometer measurements

Typical measurements of a cone penetrometer include the cone penetration resistance ${q}_{c}$, sleeve friction resistance ${f}_{s}$, and porewater pressure $u$ generated during cone penetration, ${u}_{1}$ when measured on the cone face, ${u}_{2}$ when measured just above the cone (standard location), and ${u}_{3}$ when measured just above the friction sleeve. Generally, the sleeve friction resistance is considered less accurate than the cone tip penetration resistance and cone penetration porewater pressure^2,7^.

Variations in cone penetrometer design and porewater pressure effects produce changes in the measured tip penetration resistance and sleeve friction; therefore, corrections are needed to obtain accurate and repeatable measurements using the CPT. The geometric configuration of the cone penetrometer exposes annular-shaped surfaces at the cone base edge and at the friction sleeve top and base, which are subject to porewater pressure. The action of the water pressure on these areas in the axial direction, with different intensities and senses, gives rise to normal forces on the cone base and, in the case of a friction sleeve with unequal end areas, to the resultant axial forces in the friction sleeve when acting on water or resultant tangential forces on the outer surface of the friction sleeve when acting on soil, producing additional contribution to the cone and sleeve penetration resistance. This effect on friction sleeve mobilization is commonly termed the ’unequal end area effect’^8^. Despite having unequal end areas, the cone tip does not present such a water effect. Under such circumstances, ${q}_{c}$ and ${f}_{s}$ do not correspond to the total resistance offered by the surrounding soil.

The measured cone penetration resistance ${q}_{c}$ can be corrected to the total cone penetration resistance ${q}_{t}$^8,9^ using

1 $${q}_{t}={q}_{c}+\left(1-a\right){u}_{2}$$

where $a={A}_{cs}/{A}_{cb}$ is the net area ratio, ${A}_{cs}$ is the cross-sectional area of the central shaft at the cone base, and ${A}_{cb}$ is the area of the cone base.

The measured sleeve friction resistance ${f}_{s}$ can be corrected to the total sleeve friction resistance ${f}_{t}$^10^ using

2 $${f}_{t}={f}_{s}-\left({u}_{2}{A}_{sb}-{u}_{3}{A}_{st}\right)/{A}_{s}$$

where ${A}_{sb}$ is the area of the sleeve base, ${A}_{st}$ is the area of the sleeve top, and ${A}_{s}$ is the surface area of the friction sleeve.

These effects are significant in fine-grained soils and in overwater work, essentially when high water pressure occurs. Fine-grained soils exhibit undrained behaviour during the cone penetration process, producing considerable porewater pressure. The water pressure effects become particularly important in soft soils where, due to the low penetration resistance, the porewater pressure reaches a substantial magnitude in relation to ${q}_{c}$ and ${f}_{s}$, resulting in a relevant influence on the total resistance to penetration, ${q}_{t}$ and ${f}_{t}$. When the CPT is performed with friction sleeves of unequal end areas in deep water sediments, for instance, before the cone penetrates the soil, the high water pressure unloads the sleeve friction load cell, generating a significant negative offset. Thus, the zero-load stability becomes a major issue as the ${f}_{s}$ measurements are made very close to zero load^11^. Because of the drained penetration, ${q}_{t}={q}_{c}$ in sandy soils.

Inaccurate ${f}_{s}$ measurements are attributable to the unequal end area effect, tolerance in dimensions between the cone and sleeve, surface roughness of the sleeve, and load cell design and calibration^12^. Friction sleeves with equal end areas do not produce water pressure effects, which excludes the need for any correction to ${f}_{s}$, hence providing more reliable sleeve friction values^3^. With good design (separate load cells, equal end area friction sleeve, and compensated shear load cell) and quality control (zero load measurements, tolerances, and surface roughness), it is possible to obtain repeatable sleeve friction measurements^11^. Different cone penetrometer designs produce varying friction sleeve measurements and error relevance; in general, however, the cone penetration resistance shows relatively small variation, and the penetration porewater pressure is accurate and repeatable^7,13^. The penetration porewater pressure can suffer from a lack of repeatability owing to the loss of saturation, especially when performed onshore at locations where the water table is deep and/or in very stiff soils^6^. A detailed description of the major issues related to cone design and procedures has been presented^14^.

## Normalization of cone penetrometer measurements

Experiments have shown that the resistance to cone penetration increases with depth, that is, with increasing effective confining stress^15–17^. The variation in cone measurements due to variation in the soil shear strength and stiffness with the in situ stress state affects the interpretation of soil behaviour. Therefore, to compare soil behaviour at different depths and thus provide an appropriate geotechnical prediction from cone penetrometer data, stress normalization must be applied to the cone measurements to convert them into an equivalent value at a standard atmospheric stress. The stress normalization allows a variable property to be reduced to an equivalent value at a standard confining stress, such as vertical, mean, octagonal, or lateral stress in atmospheric pressure units ($atm$) or with reference to atmospheric pressure of ${P}_{a}=1atm \left(\approx 100kPa\right)$. As the resistance to cone penetration tends to increase as the effective overburden stress increases with depth, the in situ effective vertical stress ${\sigma }_{vo}^{^{\prime}}$ can be used as a standard confining stress. The normalization of cone measurements utilizes the factor $c=1/{\left({\sigma }_{vo}^{^{\prime}}\right)}^{n}$ or $c=1/{\left({\sigma }_{vo}^{^{\prime}}/{P}_{a}\right)}^{n}$, referred to as stress exponent-based stress normalization, where *n* is the stress exponent, which defines the dependence of the measured parameter on the in situ effective vertical stress so that $n=0$ means no dependence, $n=1$ means linear dependence and $n<1$ means nonlinear dependence. This approach, originally proposed for SPT normalization^18^, has been extensively used for CPT normalization, as summarised in Table 1.

**Table 1:** Normalization of cone penetrometer measurements.

CPT normalization			References
Cone resistance	Sleeve friction	Porewater pressure	
$\frac{{q}_{c}}{{\left({\sigma }_{vo}^{^{\prime}}\right)}^{n}}$	$\frac{{f}_{s}}{{\left({\sigma }_{vo}^{^{\prime}}\right)}^{n}}$	–	^19–22,88^
$\frac{{q}_{c}-{\sigma }_{vo}}{{\left({\sigma }_{vo}^{^{\prime}}\right)}^{n}}$	–	–	^23^
${q}_{c}{\left(\frac{{P}_{a}}{{\sigma }_{vo}^{^{\prime}}}\right)}^{0.5}$	–	–	^24,25^
${q}_{c}{\left(\frac{{P}_{a}}{{\sigma }_{vo}^{^{\prime}}}\right)}^{n}$	–	–	^26,27^
${q}_{c}{\left(\frac{{P}_{a}}{{\sigma }_{vo}^{^{\prime}}}\right)}^{n}$	${f}_{s}{\left(\frac{{P}_{a}}{{\sigma }_{vo}^{^{\prime}}}\right)}^{n}$	–	^29^
$\frac{1.8{q}_{c}}{0.8+\frac{{\sigma }_{vo}^{^{\prime}}}{{P}_{a}}}$	–	–	^30^
$\frac{{q}_{c}-{\sigma }_{vo}}{{P}_{a}}{\left(\frac{{P}_{a}}{{\sigma }_{vo}^{^{\prime}}}\right)}^{n}$	–	–	^4^
$\frac{{q}_{t}-{\sigma }_{vo}}{{\sigma }_{vo}^{^{\prime}}}$	–	–	^2,31,32^
$\frac{{q}_{t}-{\sigma }_{vo}}{{\sigma }_{vo}^{^{\prime}}}$	$\frac{{f}_{s}}{{\sigma }_{vo}^{^{\prime}}}$	–	^33^
$\frac{{q}_{t}-{\sigma }_{vo}}{{\sigma }_{vo}^{^{\prime}}}$	–	$\frac{{u}_{2}-{u}_{o}}{{\sigma }_{vo}^{^{\prime}}}$	^34^
$\frac{{q}_{t}}{{P}_{a}}{\left(\frac{{P}_{a}}{{\sigma }_{vo}^{^{\prime}}}\right)}^{0.5}$	–	–	^35^
${\left({q}_{t}-{\sigma }_{vo}\right)\left(\frac{{P}_{a}}{{\sigma }_{vo}^{^{\prime}}}\right)}^{n}$	–	–	^36,37^
$\frac{{q}_{t}-{\sigma }_{vo}}{{P}_{a}}{\left(\frac{{P}_{a}}{{\sigma }_{vo}^{^{\prime}}}\right)}^{n}$	–	–	^38,39,40^

To account for the complexity of soil resistance with respect to the degree of linearity dependency on increasing vertical effective stress, several approaches to determining the stress exponent have been proposed. Olsen and Mitchell^23^ suggested a stress normalization exponent given by

3 $$n=1-0.007\left({D}_{r}-10\%\right)$$

where ${D}_{r}$ is the relative density. Boulanger and Idriss^26^ and Idriss and Boulanger^27^ recommended using

4 $$n=0.784-0.521{D}_{r}$$

Zhang et al.^28^ proposed obtaining a stress normalization exponent, expressed as

5 $$n=0.5\, if\, {I}_{c}\le 1.64$$

6 $$n=0.3\left({I}_{c}-1.64\right)+0.5\,\mathrm{ if }\,1.64<{I}_{c}<3.30$$

7 $$n=1\, if\,{I}_{c}\ge 3.30$$

8 $${I}_{c}={\left[{\left(3.47-\mathit{log}Q\right)}^{2}+{\left(1.22+\mathit{log}F\right)}^{2}\right]}^{0.5}$$

9 $$Q=\left(\frac{{q}_{c}-{\sigma }_{vo}}{{P}_{a}}\right){\left(\frac{{P}_{a}}{{\sigma }_{vo}^{^{\prime}}}\right)}^{n}$$

10 $$F=\frac{{f}_{s}}{{q}_{c}-{\sigma }_{vo}}100\%$$

and if ${\sigma }_{vo}^{^{\prime}}>300kPa$, $n=1$ for all soils. The stress exponent is determined by an iterative process starting with $n=1$ until the change in $n$, $\Delta n$, becomes less than $0.01$, $\Delta n<0.01$. ${I}_{c}$ is updated from Robertson and Wride^4^. Moss et al.^29^ suggested a stress normalization exponent for normalizing both ${q}_{c}$ and ${f}_{s}$, given by

11 $$n=\frac{0.78}{{{q}_{c}}^{0.33}}{\left(\frac{\left({f}_{s}/{q}_{c}\right)100}{abs{\left[\mathit{log}\left(10+{q}_{c}\right)\right]}^{1.21}}\right)}^{0.32{{q}_{c}}^{-0.35}-0.49}$$

Cetin and Isik^36^ developed an iterative procedure for estimating the stress normalization exponent, as defined by

12 $$n=\frac{R-272.38}{275.19-272.38}\pm 0.085, 272.38<R<275.19$$

13 $$\mathrm{R}={\left[{\left({\mathrm{logF}}_{\mathrm{R}}+234.91\right)}^{2}+{\left(\mathrm{log}\left(\frac{{\mathrm{q}}_{\mathrm{t},1,\mathrm{net}}}{{\mathrm{P}}_{\mathrm{a}}}\right)-126.24\right)}^{2}\right]}^{0.5}$$

14 $${F}_{R}=\frac{{f}_{s}}{{q}_{c}-{\sigma }_{vo}}100\%$$

15 $${q}_{t,1,net}={{q}_{t}-{\sigma }_{vo}\left(\frac{{P}_{a}}{{\sigma }_{vo}^{^{\prime}}}\right)}^{n}$$

which typically varies in the range of $0.4{-}0.65$ for sands, $0.6{-}0.8$ for sand/silt/clay mixtures, and $0.9{-}1$ for clays. Robertson^3^ proposed a stress normalization exponent given by

16 $$n=0.381{I}_{c}+0.05\left(\frac{{\sigma }_{vo}^{^{\prime}}}{{P}_{a}}\right)-0.15, n\le 1$$

17 $${I}_{c}={\left[{\left(3.47-\mathrm{log}{Q}_{t1}\right)}^{2}+{\left(1.22+\mathrm{log}{F}_{r}\right)}^{2}\right]}^{0.5}$$

18 $${Q}_{tn}=\left(\frac{{q}_{t}-{\sigma }_{vo}}{{P}_{a}}\right){\left(\frac{{P}_{a}}{{\sigma }_{vo}^{^{\prime}}}\right)}^{n}$$

19 $${F}_{r}=\frac{{f}_{s}}{{q}_{t}-{\sigma }_{vo}}100\%$$

${I}_{c}$ was adapted from Robertson and Wride^4^ based on ${Q}_{tn}$ proposed by Robertson^38^ such that $n=1$ leads to ${Q}_{tn}={Q}_{t1}$, and where ${F}_{r}$ is updated using ${q}_{t}$. Robertson^3^ assumed $n=1$ for most fine-grained soils, $n$ ranging from $0.5$ to $0.9$ for most coarse-grained soils when the in situ effective vertical stresses are not high, and $n=1$ for most soils when the in situ effective vertical stress is greater than $1MPa$. A detailed discussion on the stress normalization of cone measurements has been provided^3,34,42,43^.

The resistance to cone penetration is controlled by the pressure dependency with coupling between the volumetric and shear behaviour. The stress path or shearing condition imposed by the cone penetration process and the initial state of the soil, defined by the effective vertical stress and degree of over consolidation (normally consolidated, NC, lightly overconsolidated, LOC, and heavily overconsolidated, HOC, states), are captured by the stress normalization exponent $n$. Hence, $n$ is better designated as the stress–strain-strength parameter. It defines the variation of the effective stress state and, therefore, the effects of soil strength and deformability. The definition of the stress normalization exponent is the key factor to correctly capture the soil behavior type.

For the NC and LOC undrained penetration (i.e., positive excess porewater pressure, such as in very soft to medium stiff fine-grained soil), NC and LOC drained penetration (i.e., contractive behaviour, such as in very loose to medium dense coarse-grained soil), HOC undrained penetration at high in situ effective vertical stress (i.e., positive excess porewater pressure, such as in stiff fine-grained soil), and HOC drained penetration at high in situ effective vertical stress where dilatancy is suppressed and grain crushing or breakage occurs (i.e., contractive behaviour, such as in dense coarse-grained soil, at approximately $2MPa$ for uniform silica sands, $1MPa$ for angular silica sands and silty sands, and $0.1MPa$ for carbonate sands), the total cone penetration resistance increases linearly with increasing initial effective vertical stress, such that $n=1$. However, for the HOC undrained penetration (i.e., negative excess porewater pressure, such as in stiff fine-grained soil) and HOC drained penetration (i.e., dilative behaviour, such as in dense coarse-grained soil), both at not high in situ effective vertical stress, it increases nonlinearly, and $0.5\le n<1$.

In summary, a linear stress–strain-strength behaviour ($n=1$) is assumed for soils with low to medium shear strength at failure, when positive excess porewater pressure (reduction of effective stress state; loss of shearing strength) in undrained penetration or contractive behaviour in drained penetration occurs, and a nonlinear stress–strain-strength behaviour ($0.5\le n<1$) is assumed for soils with high shear strength at failure, when negative excess porewater pressure (increase of effective stress state; gain of shearing strength) in undrained penetration or dilative behaviour in drained penetration occurs.

**Figure 1:**
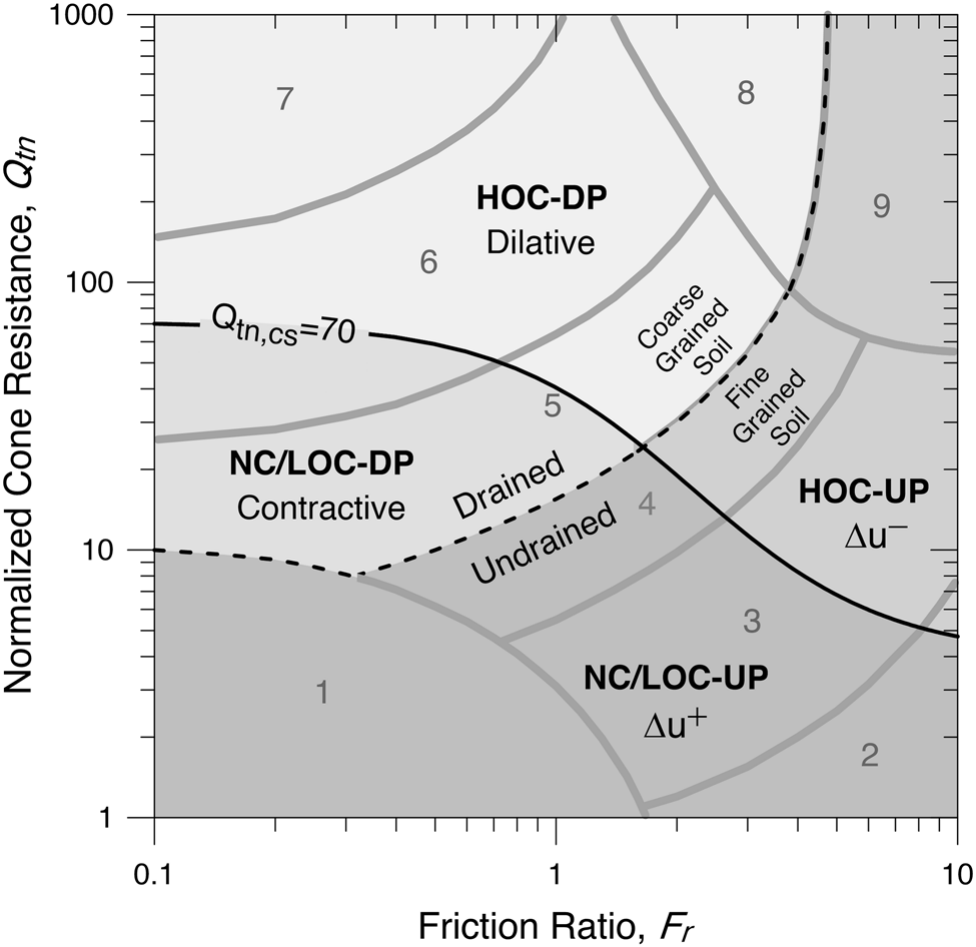
Zones of soil behaviour: NC/LOC-UP: undrained penetration into normally consolidated or lightly overconsolidated soil. NC/LOC-DP: drained penetration into normally consolidated or lightly overconsolidated soil. HOC-UP: undrained penetration into heavily overconsolidated soil. HOC-DP: drained penetration into heavily overconsolidated soil. (Adapted from Robertson^44^).

Thus, such conditions, as depicted in Fig. 1, should guide the determination of the stress normalization exponent. In general, regardless of the strain level, NC and LOC soils exhibit linear mechanical behaviour and HOC soils, highly nonlinear mechanical behaviour. The critical state line (CSL) defines the transition between the LOC and HOC states. Thus, the threshold condition for the LOC state, ${YSR}_{CSL}={\left(2/\mathrm{cos}{\phi }_{cs}\right)}^{1.25}$, irrespective of drainage condition, corresponds to $YSR\cong 2.48 \sim 2.85$ for fine-grained soils and $YSR\cong 2.85 \sim 3.32$ for coarse-grained soils, where $YSR$ is the yield stress ratio and ${\phi }_{cs}$ is the critical state friction angle. Nonetheless, as the HOC soil is characterized by an in situ effective horizontal stress greater than the in situ effective vertical stress, the transition condition between the LOC and HOC states can be more rationally defined by the coefficient of earth pressure at rest, ${K}_{o}$, such that the NC and LOC states are set for ${K}_{o}\le 1$ and HOC state for ${K}_{o}>1$, for all soils. Hence, the threshold condition for the LOC state corresponds to $YSR\cong 3.3 \sim 4$ for fine-grained soils and $YSR\cong 4 \sim 5$ for coarse-grained soils.

The clean sand equivalent penetration resistance ${Q}_{tn,cs}$ ^4^ is determined by

20 $${Q}_{tn,cs}={K}_{c}{Q}_{tn}$$

21 $${K}_{c}=1\, if\,{I}_{c}\le 1.64$$

22 $${K}_{c}=-0.403{I}_{c}^{4}+5.581{I}_{c}^{3}-21.63{I}_{c}^{2}+33.75{I}_{c}-17.881\, if\,{I}_{c}>1.64$$

where ${K}_{c}$ is a correction factor for correcting the normalized total cone penetration resistance, ${Q}_{tn}$, in silty sands to an equivalent clean sand value, ${Q}_{tn,cs}$. The contour of ${Q}_{tn,cs}=70$ defines the transition of the soil volume change or excess porewater pressure change, as illustrated in Fig. 1^44^. The region represented by ${Q}_{tn,cs}\le 70$, referring to soils that undergo contraction or positive excess porewater pressure during cone penetration, corresponds to $n=1$, and the region defined by ${Q}_{tn,cs}>70$, for soils that undergo dilation or negative excess porewater pressure during cone penetration, corresponds to $0.5\le n<1$. Under HOC penetration at high effective confining stress, the soil undergoes contraction due to grain breakage in drained shear or positive excess porewater pressure in undrained shear, leading to loss of shearing strength. It is possible that such a condition has already been covered by the region defined by ${Q}_{tn,cs}\le 70$; however, this has not yet been explicitly stated.

Therefore, the proposed guidelines for determining the stress normalization exponent are summarized as $n=1$ if ${Q}_{tn,cs}\le 70$, and $0.5\le n<1$ if ${Q}_{tn,cs}>70$, using appropriate equation for $n$.

Based on critical state concepts, Robertson^45^ proposed a boundary between contractive and dilative behaviour for Robertson ${Q}_{tn}$–${F}_{r}$ chart^2,3^ using the state parameter $\Psi$ ($\Psi =-0.05$), which confirms such boundary based on ${Q}_{tn,cs}$ (${Q}_{tn,cs}=70$). The contours of $\Psi$ and the contours of ${Q}_{tn,cs}$ present strong similarity given by

23 $$\Psi =0.56-0.33\mathrm{log}{Q}_{tn,cs}$$

Robertson^6^ defined states of soil behaviour based on the Robertson ${Q}_{tn}$–${F}_{r}$ chart^2,3^, which can also guide the determination of the stress normalization exponent. The soil states are established by the contractive-dilative parameter $CD$ and by the modified soil behaviour type index ${I}_{B}$ derived from a hyperbolic-shaped soil-type boundary suggested by Schneider et al.^5^, expressed as

24 $$CD=\left({Q}_{tn}-11\right){\left({1+0.06F}_{r}\right)}^{17}$$

25 $${I}_{B}=\frac{100\left({Q}_{tn}+10\right)}{\left(70+{Q}_{tn}{F}_{r}\right)}$$

Similar to ${Q}_{tn,cs}=70$, the contour of $CD=70$ defines the boundary of the soil volume change or excess porewater pressure change, and the contours of ${I}_{B}$ define boundaries between drained, undrained, and partial drainage conditions. The transitional zone included in the chart is related to mixed soils, where the prevailing behaviour of soil volume change or excess porewater pressure change is largely dependent on the degree of drainage as well as on the different soil components that control its mechanical behaviour. Hence, the transitional zone becomes an uncertainty zone for interpreting soil behaviour. Thus, by focusing on the prediction of soil behaviour type rather than soil identification, a value of ${I}_{B}$ between $22$ and $32$ that defines a contour between drained and undrained conditions must exist, as defined by Robertson^44^.

## Cone penetrometer-based soil type classification

In the systematic categorization of soil type based on cone penetrometer testing, soils are classified into groups that exhibit similar mechanical behaviour, not by the physical characteristics of the soil constitution. Conventionally, soil type identification is obtained from laboratory testing performed on disturbed samples, usually by grain size distribution and Atterberg limits (e.g., Unified Soil Classification System, USCS). Fortunately, there is reasonable agreement between the CPT-based SBT and USCS-based soil classification^1,46,47^. This is verified for penetration in sand and clay that typically occur under fully drained or undrained conditions, respectively. The largest difference is likely to occur for mixed soils (i.e., sand mixtures and silt mixtures), where the degree of drainage and different components of the soils control different aspects of the mechanical behaviour of these soils^1,3^. Penetration in mixed soils is conducted under partial drainage, and the resistance to penetration is controlled by the degree of porewater pressure^34^. The soil composition and mineralogy; type, content, and plasticity of fines; stress history; and stress level also produce different soil responses. Several examples of the differences that can arise in mixed soils have been reported^3^. Ideally, a classification system with a strong link to in situ behaviour is desirable for geotechnical engineering purposes; however, a combined classification based on both physical and behavioural characteristics would be helpful^48^.

Comprehensive experience exists concerning the soil type classification from the CPT and CPTu data. Begemann^49^ pioneered CPT-based soil type prediction from mechanical cone data, presenting a linear relation between ${q}_{c}$ and ${f}_{s}$, as fanned-out lines from the origin with an angular coefficient, ${q}_{c}/{f}_{s}$, showing that the soil type is a function of the friction ratio, ${R}_{f}=\left({f}_{s}/{q}_{c}\right)100\%$. Subsequent studies employed ${R}_{f}$ in soil type interpretation^49,50^ or from ${q}_{c}$ and ${R}_{f}$ relations^1,41,51–57^.

Insights into the cone penetration process have shown the need to account for the correction and normalization of cone measurements. Thus, extensive use is made of the corrected and normalized ($\check{n}$) cone parameters for soil type predictions based on ${q}_{c,\check{n}}$ and ${f}_{s,\check{n}}$^19,20^, ${q}_{c,\check{n}}$ and ${R}_{f,\check{n}}$^21–23,42^, ${q}_{t}$ and ${f}_{s}$^58^, ${q}_{t}$ and ${R}_{f}$^59^, ${q}_{t,\check{n}}$ and ${f}_{s,\check{n}}$^33^, ${q}_{t,\check{n}}$ and ${u}_{2,\check{n}}$^34^, ${q}_{t,\check{n}}$ and ${F}_{R}$^36,37^, and ${q}_{t,\check{n}}$ and ${F}_{r}$^2,3,5,6^.

Recognizing the problems associated with sleeve friction measurements, several soil classification methods have been proposed based on cone penetration resistance and penetration porewater pressure, which are considered more reliable measurements. Initially, ${q}_{c}$ and ${u}_{2}$ are used for soil-type identification^60,61^. The porewater pressure ratio ${B}_{q}=\left({u}_{2}-{u}_{o}\right)/\left({q}_{t}-{\sigma }_{vo}\right)$ was proposed^62^, and soil type classification charts based on ${q}_{t}$ and ${B}_{q}$ have been presented^2,5,34,54,59,62–65^. The effective porewater pressure ratio ${B}_{E}=\Delta {u}_{2}/{u}_{o}$ was suggested along with a soil-type chart based on ${q}_{t}$ and ${B}_{E}$, where $\Delta {u}_{2}={u}_{2}-{u}_{o}$ is the excess penetration porewater pressure^66^. A chart for identifying soil behaviour type using ${q}_{t}/{u}_{o}$ and ${B}_{p}=\Delta {u}_{2}/\left({q}_{t}-{u}_{o}\right)$ ^67^ and a chart that relates normalized cone tip resistance ${Q}_{t1}$ and normalized excess porewater pressure $\Delta {u}_{2}/{\sigma }_{vo}^{^{\prime}}$ as a function of the overconsolidation ratio^5,34^ were also proposed. $\Delta {u}_{2}/{\sigma }_{vo}^{^{\prime}}$ is considered a better form of the porewater pressure parameter than ${B}_{q}$ ^34^.

Some proposals for classifying soil behaviour types have gathered all basic cone measurements. SBTn charts were developed by considering ${Q}_{t1}\left(1-{B}_{q}\right)$ ^69,70^ or ${Q}_{t1}\left(1-{B}_{q}+1\right)$ ^68^ and ${F}_{r}$ , where ${Q}_{t1}\left(1-{B}_{q}\right)+1$ is a normalized effective cone resistance that can be rewritten as $\left({q}_{t}-{u}_{2}\right)/{\sigma }_{vo}^{^{\prime}}$. A soil characterization chart was proposed based on nonnormalized parameters, using ${q}_{t}-{u}_{2}$ and ${f}_{s}$, where ${q}_{e}={q}_{t}-{u}_{2}$ is the effective cone resistance^71^. The charts based on the effective cone resistance suffer from a lack of accuracy in soft fine-grained soils, where ${q}_{t}$ is small compared with ${u}_{2}$ and the difference, ${q}_{t}-{u}_{2}$, becomes very small^3^. In addition, ${q}_{e}$ does not correspond to the effective cone resistance available at the cone tip, because the maximum porewater pressure is mobilized at the cone face (*u*_1_ position)^72–75^, not at the cone base (*u*_2_ position).

Several algorithms have been proposed for the classification of soil types using probabilistic methods^37,76^, fuzzy logic^77,78^, artificial neural networks^79–82^, and machine learning^83,84^.

Emphasis is given to the soil behaviour type classification system developed by Robertson^2,3^, using ${Q}_{tn}$ and ${F}_{r}$, and modified by Robertson^85^ using nonnormalized basic cone parameters ${q}_{c}/{p}_{a}$ and ${R}_{f}$.

Based on works by Wroth^31,86^ and Houlsby^32^, Robertson^2^ suggested consistent soil behaviour type charts, ${Q}_{t}$–${F}_{r}$ and ${Q}_{t}$–${B}_{q}$, but recommended the ${Q}_{t}$–${F}_{r}$ chart as generally more reliable, where

26 $${Q}_{t}=\frac{{q}_{t}-{\sigma }_{vo}}{{\sigma }_{vo}^{^{\prime}}}=\frac{{q}_{n}}{{\sigma }_{vo}^{^{\prime}}}$$

27 $${F}_{r}=\frac{{f}_{s}}{{q}_{t}-{\sigma }_{vo}}100\%=\frac{{f}_{s}}{{q}_{n}}100\%$$

28 $${B}_{q}=\frac{{u}_{2}-{u}_{o}}{{q}_{t}-{\sigma }_{vo}}=\frac{\Delta {u}_{2}}{{q}_{n}}$$

and ${q}_{n}={q}_{t}-{\sigma }_{vo}$ is the net cone penetration resistance.

The magnitude of the excess porewater pressure depends on the shearing process and changes in the total stress induced by cone penetration. In an undrained situation, the shear stress can only be expressed in terms of principal stresses as the difference between two total stresses or a difference of two effective stresses. As the cone penetration resistance is a measure of the shear strength of the soil in terms of the total stress, the variable ${q}_{n}={q}_{t}-{\sigma }_{vo}$ is a measure of the maximum shear stress. Thus, the parameter ${B}_{q}$ is a ratio of the excess porewater pressure to the shear stress at failure, ${F}_{r}$ is a ratio of the sleeve friction resistance to the shear stress at failure, and ${Q}_{tn}$ is the shear stress at failure normalized as a function of the effective vertical stress. ${F}_{r}$ and ${B}_{q}$ are not required for stress normalization because they remain constant irrespective of the stress level.

Robertson and Wride^4^ proposed a soil behaviour type index by approximating the Robertson ${Q}_{t}$–${F}_{r}$ chart^2^ as concentric circles, expressed as

29 $${I}_{c}={\left[{\left(3.47-\mathrm{log}{Q}_{tn}\right)}^{2}+{\left(1.22+\mathrm{log}{F}_{r}\right)}^{2}\right]}^{0.5}$$

replacing ${Q}_{t}$ with ${Q}_{tn}$, given by

30 $${Q}_{tn}=\left(\frac{{q}_{t}-{\sigma }_{vo}}{{P}_{a}}\right){\left(\frac{{P}_{a}}{{\sigma }_{vo}^{^{\prime}}}\right)}^{n}$$

as suggested by Robertson^38^. Robertson^3^ updated the method to define the normalized stress exponent as

31 $$n=0.381{I}_{c}+0.05\left(\frac{{\sigma }_{vo}^{^{\prime}}}{{P}_{a}}\right)-0.15$$

where $n\le 1$. The approximation suggested by Robertson and Wride^4^ was disadvantageous for predicting the behaviour of mixed and fine-grained soils compared to the original method of Robertson^2^. The hyperbolic-shaped soil-type boundaries suggested by Schneider et al.^5^ also significantly differ from the Robertson^2,3^ SBTn zones.

Robertson^85^ presented a nonnormalized SBT chart, where the boundaries are also concentric circles and a nonnormalized soil behaviour type index, ${I}_{SBT}$, is given by

32 $${I}_{SBT}={\left[{\left(3.47-\mathrm{log}\left(\frac{{q}_{c}}{{P}_{a}}\right)\right)}^{2}+{\left(1.22+\mathrm{log}{R}_{f}\right)}^{2}\right]}^{0.5}$$

The nonnormalized SBT index ${I}_{SBT}$ is essentially the same as the normalized SBTn index ${I}_{c}$ but only uses the basic CPT measurements. Robertson^85^ stressed that the normalized ${I}_{c}$ generally provides a more reliable identification of SBT than the nonnormalized ${I}_{SBT}$. However, when the in situ effective vertical stress is between $50kPa$ and $150kPa$, there is often little difference between normalized and nonnormalized SBTs.

## Proposed soil behaviour type indexes

The CPT-based soil behaviour type method, ${Q}_{tn}$–${F}_{r}$ SBTn chart, proposed by Robertson^2,3^, can be expressed as concentric logarithmic spirals given by

33 $$\theta \mathrm{cot}\alpha -\mathrm{log}\left(\frac{r}{{r}_{o}}\right)=0$$

where $\theta$ is the angle of rotation as the curve spirals, $r$ is the radius vector measured from the origin to a point on the spiral, ${r}_{o}$ is the initial radius vector for $\theta =0^\circ$, and $\alpha$ is the angle between the radial and tangent lines at the point ($r,\theta$) on the spiral.

**Figure 2:**
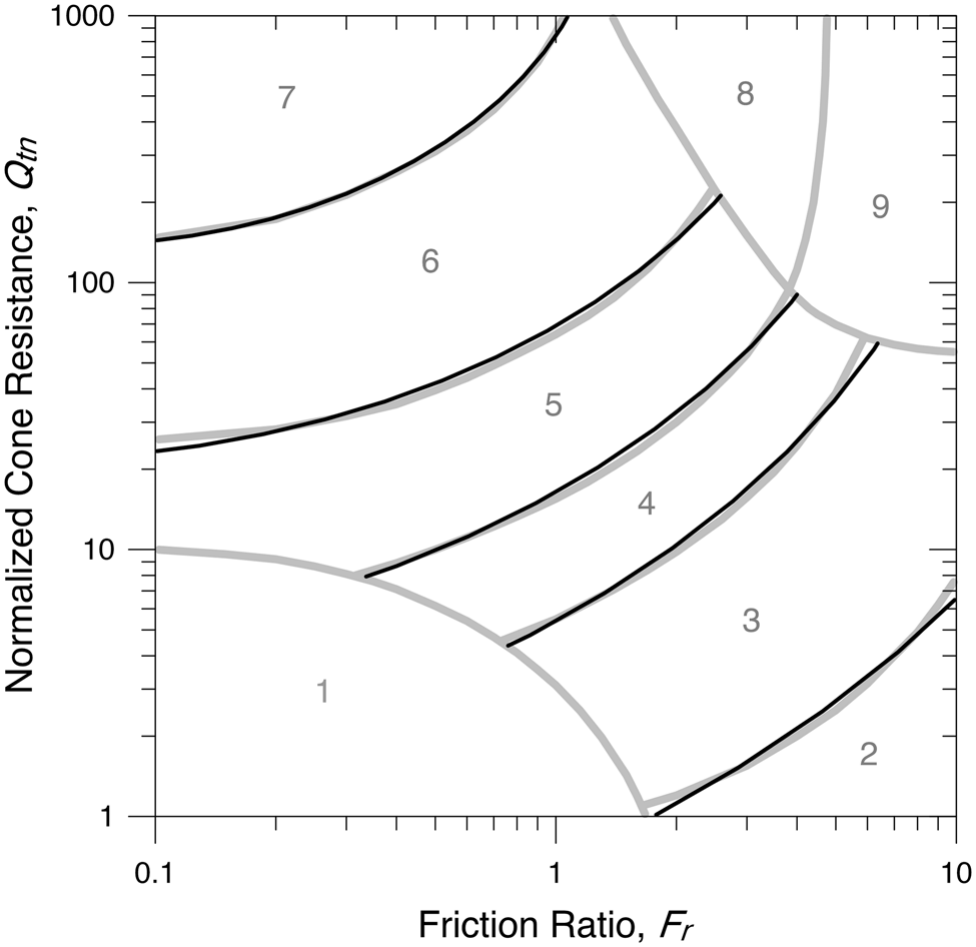
Contours of the logarithmic spirals that best fit the SBTn zone boundaries.

The boundaries between SBTn zones 2 to 7 of the chart can be individually approximated as a natural logarithmic function from Eq. (33). The position of the SBTn chart on the Cartesian plane used by Robertson and Wride^4^ was assumed as a reference and is denoted herein as RW. The best fit is given in Table 2 and shown in Fig. 2.

**Table 2:** Parameters of the logarithmic spirals that best fit the Robertson^2,3^ SBTn zone contours.

Interface	2–3	3–4	4–5	5–6	6–7
${r}_{o}$	4.22	3.37	2.84	2.13	1.33
$\alpha$ (degrees)	105.90	105.58	101.64	93.94	89.76
${R}^{2}$	0.945	0.976	0.971	0.909	0.990

However, to obtain a soil behaviour type index, a single approximation covering all soil zones is required. Inevitably, an overall approximation reduces the quality of the fit at the soil zone interface compared to that obtained independently. The initial radius of the logarithmic spiral at each soil zone contour can then be used as a soil behaviour type index.

Thus, by seeking an analytical solution, a new SBTn index can be defined as

34 $${I}_{c,RW}={e}^{\frac{\mathrm{ln}\left(\sqrt{{f}^{2}+{q}^{2}}\right)-0.106{\mathrm{tan}}^{-1}\left(\frac{f}{q}\right)}{1-{0.283\mathrm{tan}}^{-1}\left(\frac{f}{q}\right)}}$$

where $$f=1.22+\mathrm{log}{F}_{r}$$ and $$q=3.47-\mathrm{log}{Q}_{tn}$$.

The normalized cone parameter ${Q}_{tn}$ is estimated using

35 $$n=0.2969{I}_{c,RW}+0.05\left(\frac{{\sigma }_{vo}^{^{\prime}}}{{P}_{a}}\right)-0.0208$$

where $n\le 1$ and $n$ are calculated using ${I}_{c,RW}$ from ${Q}_{tn}={Q}_{t1}$ based on $n=1$.

**Figure 3:**
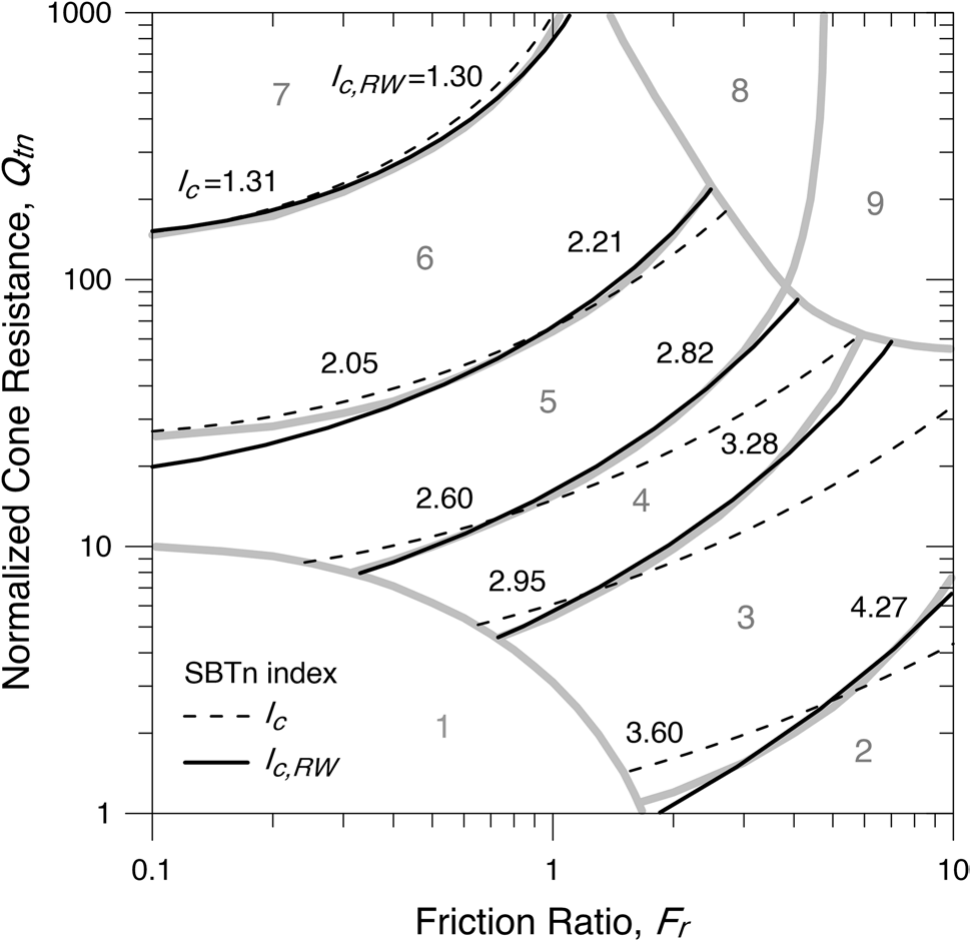
Contours of the SBTn zones of ${I}_{c}$ and ${I}_{c,RW}$.

The boundaries of the soil behaviour type index, ${I}_{c,RW}$, are shown in Fig. 3.

By adjusting the SBTn chart on a different scale, a better analytical solution can be obtained by

36 $${I}_{cs}={e}^{\frac{\mathrm{ln}\left(\sqrt{{f}^{2}+{q}^{2}}\right)-0.0884{\mathrm{tan}}^{-1}\left(\frac{f}{q}\right)}{1-{0.075\mathrm{tan}}^{-1}\left(\frac{f}{q}\right)}}$$

where $$f=1.5+1.5\mathrm{log}{F}_{r}$$ and $$q=4-\mathrm{log}{Q}_{tn}$$.

The normalized cone parameter ${Q}_{tn}$ is estimated using

37 $$n=0.3316{I}_{cs}+0.05\left(\frac{{\sigma }_{vo}^{^{\prime}}}{{P}_{a}}\right)-0.2319$$

where $n\le 1$ and $n$ are calculated using ${I}_{cs}$ from ${Q}_{tn}={Q}_{t1}$ based on $n=1$.

**Figure 4:**
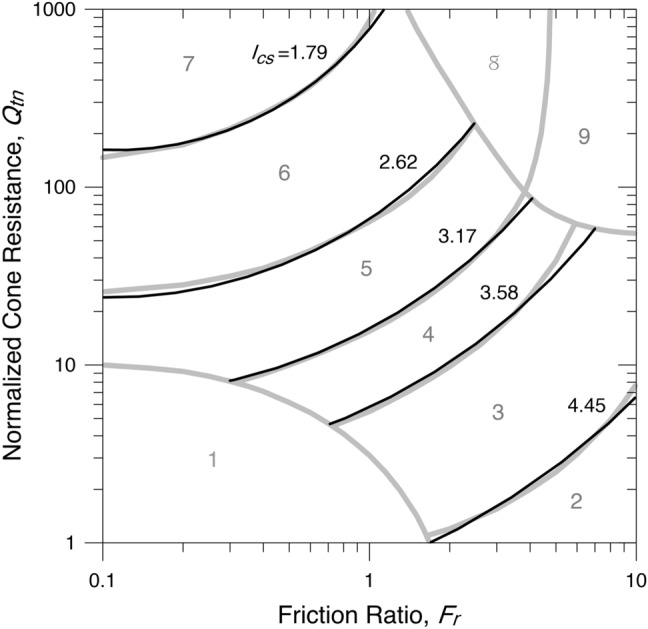
Contours of the SBTn zones of ${I}_{cs}$.

The additional subscript $s$ in ${I}_{cs}$ refers to the initial of spiral concerning the fit by logarithmic spirals. The boundaries of the soil behaviour type index, ${I}_{cs}$, are shown in Fig. 4 and given in Table 3.

**Table 3:** Boundaries of the SBTn zones of ${I}_{cs}$.

Zone	Soil behaviour type	${I}_{cs}$
1	Sensitive, fine-grained	n/a
2	Organic soils–clay	> 4.45
3	Clays–silty clay to clay	3.58–4.45
4	Silt mixtures–clayey silt to silty clay	3.17–3.58
5	Sand mixtures–silty sand to sandy silt	2.62–3.17
6	Sands–clean sand to silty sand	1.79–2.62
7	Gravelly sand to dense sand	< 1.79
8	Very stiff sand to clayey sand*	n/a
9	Very stiff, fine-grained*	n/a

*Heavily overconsolidated or cemented.

Alternatively, an accurate approximation can be achieved through natural exponential functions given by

38 $${Q}_{tn}=\lambda {e}^{\zeta {F}_{r}}$$

that vary according to

39 $$\lambda =23.283{\zeta }^{2.422} \left({R}^{2}=0.9866\right)$$

so that

40 $${F}_{r}\zeta -\mathrm{ln}{Q}_{tn}+2.422\mathrm{ln}\zeta +3.148=0$$

Therefore, a numerical root-finding method can be used to compute $\zeta$ from Eq. (40). The Newton–Raphson method converges very fast to $\zeta$. Applying the procedure at the boundaries between zones 2 to 7 corresponds to a soil behaviour type index so that it is renamed ${I}_{ce}$. The additional subscript $e$ in ${I}_{ce}$ refers to the Euler number concerning the fit by natural exponential functions. Because $\zeta$ varies linearly with $\lambda$ locally at each interface, a simple mean is assumed for ${I}_{ce}$ as the best fit.

The normalized cone parameter ${Q}_{tn}$ is estimated using

41 $$n=-0.4214\mathrm{ln}{I}_{ce}+0.05\left(\frac{{\sigma }_{vo}^{^{\prime}}}{{P}_{a}}\right)+0.6369$$

where $n\le 1$ and $n$ are calculated using ${I}_{ce}$ from ${Q}_{tn}={Q}_{t1}$ based on $n=1$.

**Figure 5:**
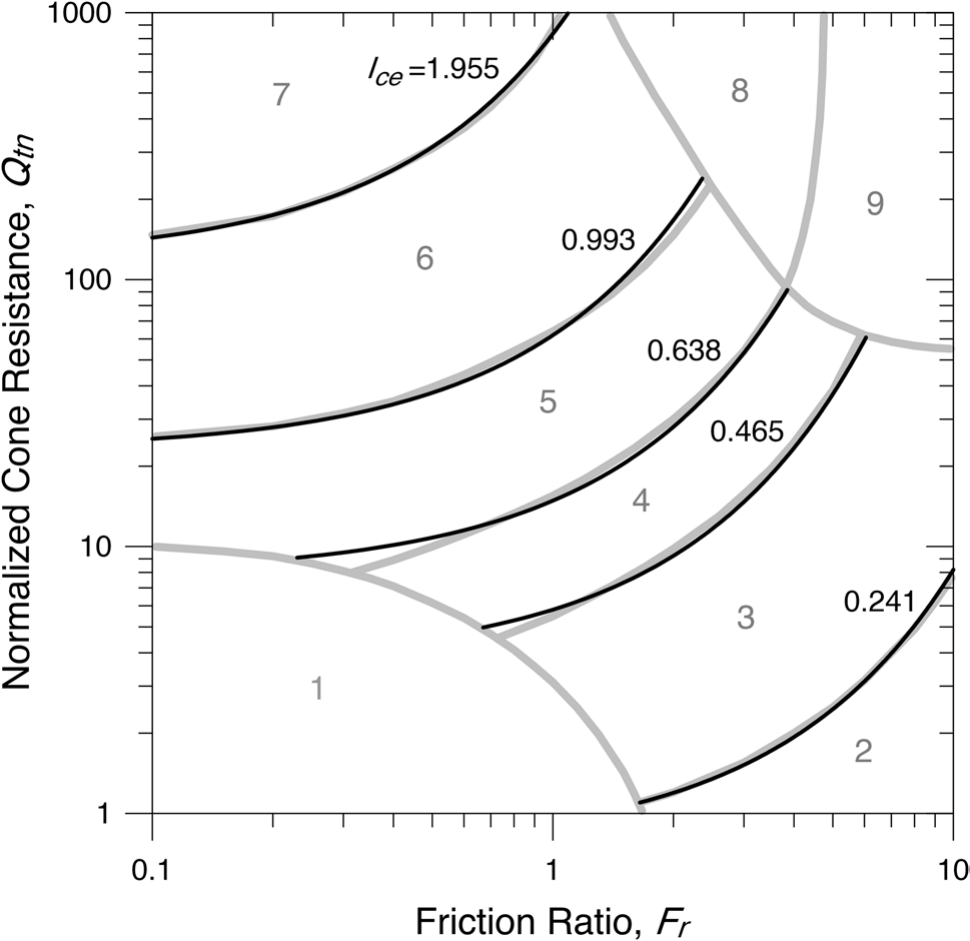
Contours of the SBTn zones of ${I}_{ce}$.

The boundaries of the soil behaviour type index, ${I}_{ce}$, are shown in Fig. 5 and given in Table 4.

**Table 4:** Boundaries of the SBTn zones of ${I}_{ce}$.

Zone	Soil behaviour type	${I}_{ce}$
1	Sensitive, fine-grained	n/a
2	Organic soils–clay	< 0.241
3	Clays–silty clay to clay	0.241–0.465
4	Silt mixtures–clayey silt to silty clay	0.465–0.638
5	Sand mixtures–silty sand to sandy silt	0.638–0.993
6	Sands–clean sand to silty sand	0.993–1.955
7	Gravelly sand to dense sand	> 1.955
8	Very stiff sand to clayey sand*	n/a
9	Very stiff, fine-grained*	n/a

*Heavily overconsolidated or cemented.

Conversely,

42 $$\zeta =0.276{\lambda }^{0.407} \left({R}^{2}=0.9866\right)$$

so that

43 $$0.276{F}_{r}{1.503}^{\mathrm{ln}\lambda }-\mathrm{ln}{Q}_{tn}+\mathrm{ln}\lambda =0$$

The approximate limits of the SBTn zones can be obtained by computing $\lambda$ from Eq. (43) using a root-finding method, giving rise to a new SBTn index. For simplicity, the results of this index are not presented here because it is an equivalent solution to that of Eq. (40).

The soil behaviour type indexes, ${I}_{cs}$ and ${I}_{ce}$, do not apply to zones 1, 8, and 9. They are in general agreement with the soil classification chart proposed by Robertson^2,3^ and are of easy computational implementation for post-processing results.

Once the nonnormalized SBT zones are essentially the same as the normalized SBTn ones, a new nonnormalized index, ${I}_{sbt}$, can be obtained from Eqs. (36), (40), or (43), by replacing ${Q}_{tn}$ with ${q}_{c}/{P}_{a}$ and ${F}_{r}$ with ${R}_{f}$.

## Comparing SBTn indexes in a soil profile case

Cone penetration tests are valuable tools for reliable stratigraphic soil logging. Near-continuous records with depth provide a detailed soil profile where very thin soil layers can be detected. The cone resistance is influenced by the soil ahead and behind the cone tip, depending on the strength–stiffness condition of the soil and the in situ effective confining stress^53^. It can be inferred that the zone of influence^87^ extends up to 15 cone diameters above and below the cone in denser or stiffer soils, which dilate or undergo negative excess porewater pressure during penetration (HOC soil at low effective confining stress), whereas this zone is quite small, approximately one cone diameter, in looser or softer soils that contract or undergo positive excess porewater pressure during penetration (NC and LOC soils, and HOC soil at high effective confining stress). The zone of influence above and below the cone during penetration will influence the cone resistance at the boundary between two soil types with significantly different strengths and stiffnesses, making it difficult to identify the transition between these soils^3^. Profiles of an SBTn index can provide a simple means of identifying and removing these transition zones.

The new normalized soil behaviour type indexes, ${I}_{cs}$ and ${I}_{ce}$, proposed herein, are compared to the ${I}_{c}$ index proposed by Robertson^3^ based on Robertson and Wride^4^ by applying them in a soil stratigraphy case example.

The test site is a sedimentary soil deposit located in the city of Belém in the northern region of Brazil, at $1^\circ {26}^{^{\prime}}{11}^{^{\prime\prime}}S$ and $48^\circ {28}^{^{\prime}}{38}^{^{\prime\prime}}W$. The soil profile consists of a $32m$ thick fluvic deltaic deposit of normally consolidated clay, intersected at a relatively deep level by a type of stone line consisting of a small, highly resistant, cemented sand mixture layer, approximately $1m$ thick, covering a large part of the subsoil of the city centre.

**Figure 6:**
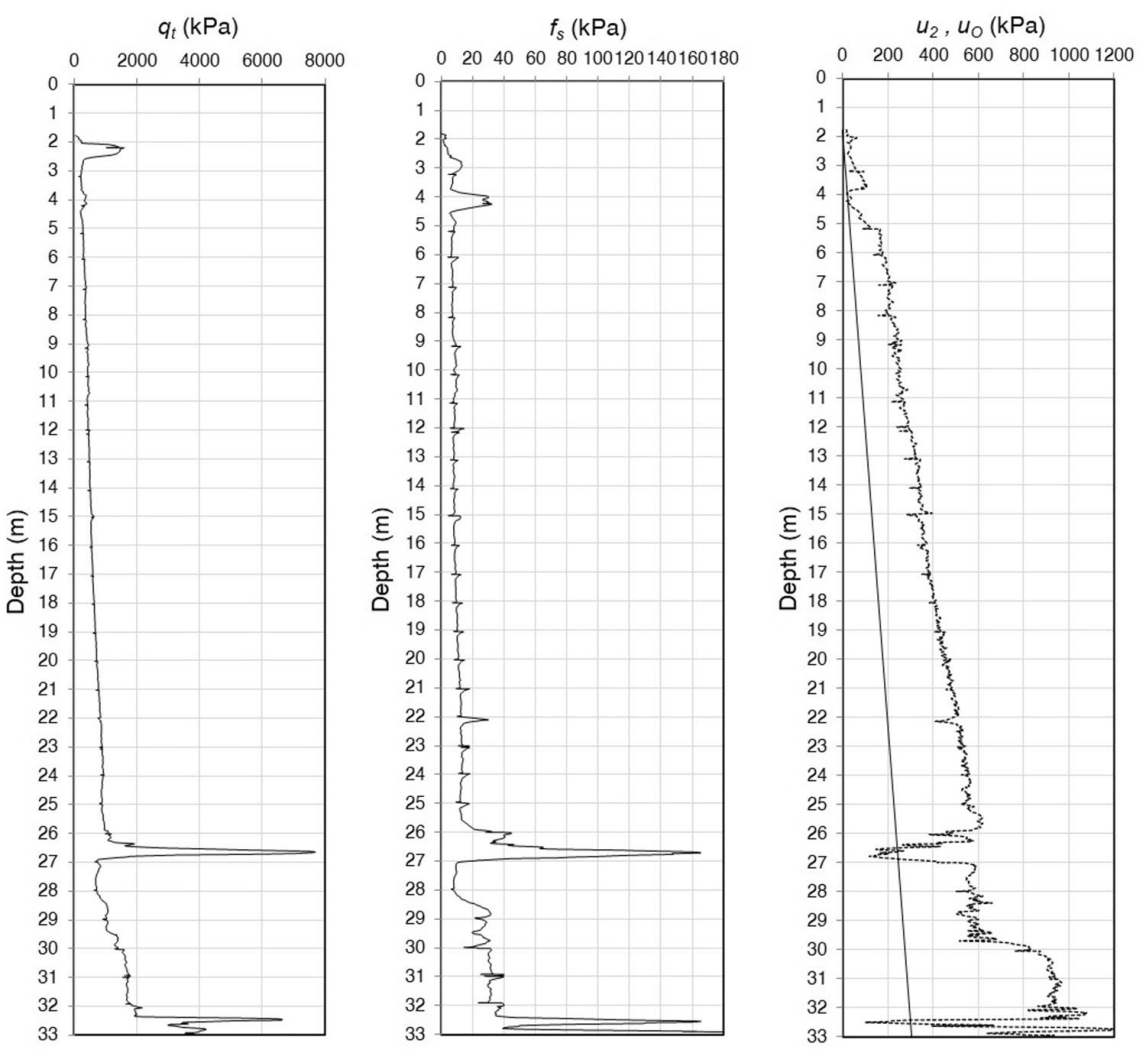
Piezocone test results from the Belém test site.

**Figure 7:**
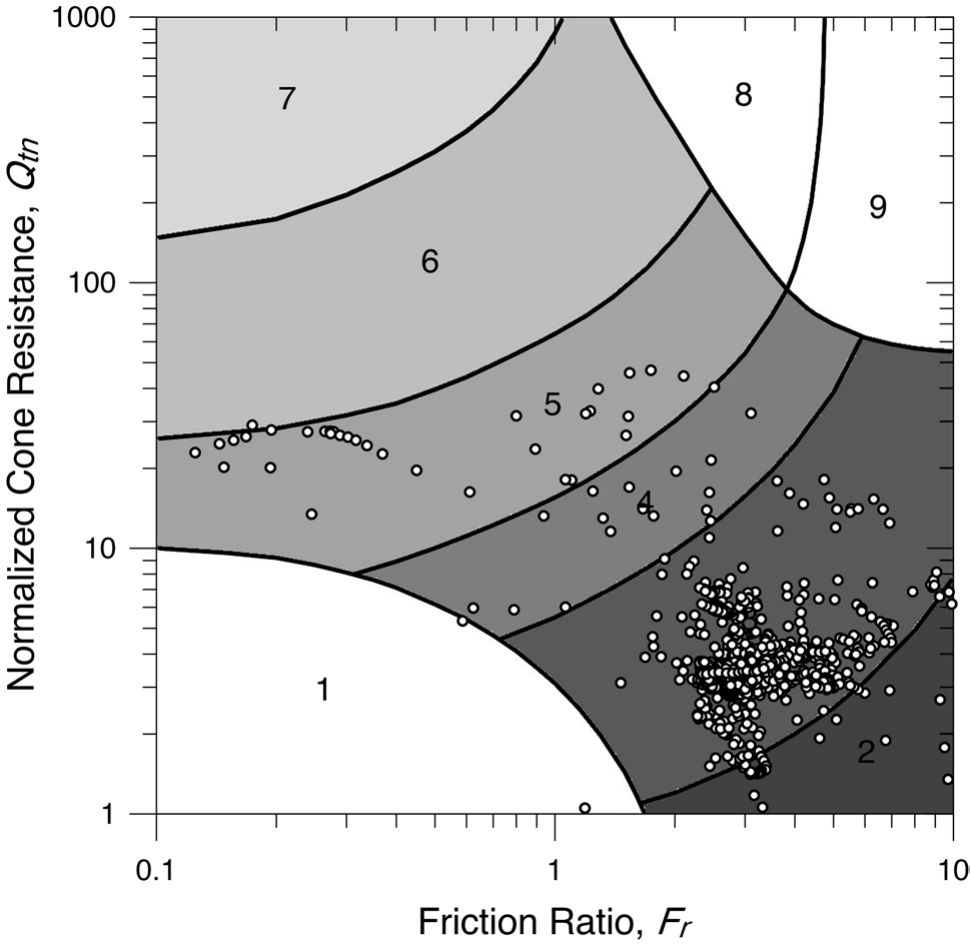
Piezocone data from the Belém test site depicted in the Robertson^3^ SBTn chart.

**Figure 8:**
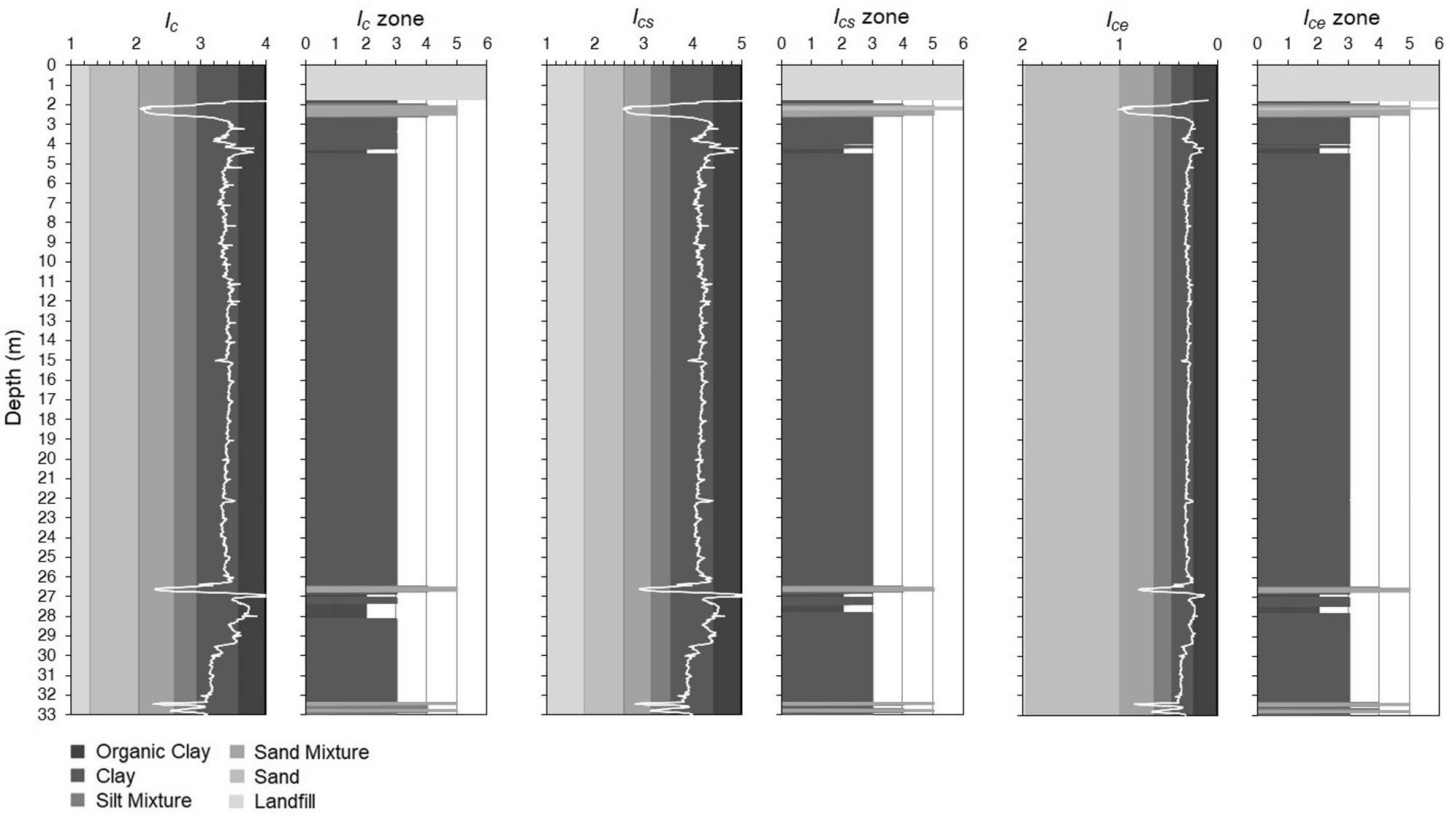
Soil stratigraphic profiling of the Belém test site from the CPT-based SBTn indexes.

The total cone penetration resistance, sleeve friction resistance, and penetration porewater pressure are presented in Fig. 6. The piezocone test results are depicted in the ${Q}_{tn}$–${F}_{r}$ chart^3^, as shown in Fig. 7, to illustrate their overall distribution in the soil behaviour type zones, mainly their position relative to the zone boundaries. The soil profile of the test site determined from the soil behaviour type indexes is shown in Fig. 8. The results illustrate the differences in the soil type prediction of both fine-grained and coarse-grained soils.

## Conclusions

Supported by strong theoretical and experimental knowledge, the cone penetrometer test has become an accurate, thorough and reliable tool for predicting soil behaviour type. Significant research into soil-type classification based on CPT and CPTu exists. Rational interpretations consider the correction and normalization of cone measurements. The cone parameter is normalized using a stress normalization factor that defines the stress–strain-strength behaviour of the soil, which varies linearly in soils that undergo contraction or positive excess porewater pressure during penetration or nonlinearly when undergoing dilation or negative porewater pressure during penetration. The ${Q}_{tn}$–${F}_{r}$ chart proposed by Robertson^2,3^ has proven to be a consistent solution that is used extensively worldwide. However, the approximation from the SBTn index, ${I}_{c}$, proposed by Robertson and Wride^4^, significantly reduced the quality of the original method. The new SBTn indexes, ${I}_{cs}$ and ${I}_{ce}$, proposed herein, preserve the robustness of the Robertson^2,3^ method. For direct comparison among the concentric circle solution given by Robertson and Wride^4^, the ${I}_{c}$ index, and the proposed concentric logarithmic spiral solution, the ${I}_{c,RW}$ index, the chart of Robertson^2,3^ was placed in the same position on the Cartesian plane assumed by Robertson and Wride^4^ to obtain ${I}_{c,RW}$. ${I}_{c,RW}$ already shows a significant improvement relative to ${I}_{c}$; however, it is still not the best solution to be obtained by fitting with concentric logarithmic spirals. A better analytical solution can then be achieved by the ${I}_{cs}$ index by adjusting the soil classification chart at a different scale. Alternatively, the SBTn index, ${I}_{ce}$, was derived from a numerical approach based on an exponential function approximation. The new soil behaviour type indexes are in general agreement with the soil classification chart suggested by Robertson^2,3^. The ${I}_{cs}$ index is quite accurate and easy to use, but the ${I}_{ce}$ index is the best approximation solution achieved thus far.

## Data Availability

The datasets generated during and/or analysed during the current study are available from the corresponding author on reasonable request.
